# Quantitative and qualitative analysis of construction and demolition waste in Yazd city, Iran

**DOI:** 10.1016/j.dib.2018.10.141

**Published:** 2018-10-30

**Authors:** Mohsen Ansari, Mohammad Hassan Ehrampoush

**Affiliations:** a Student Research Committee, Shahid Sadoughi University of Medical Sciences, Yazd, Iran; b Environmental Sciences and Technology Research Center, Department of Environmental Health Engineering, Shahid Sadoughi University of Medical Sciences, Yazd, Iran

**Keywords:** Construction waste, Demolition waste, Waste composition

## Abstract

Construction and demolition waste is the major category of municipal solid waste that is important due to high volume and mass produced. Therefore, this study aims to analyze the quantitative and qualitative of construction and demolition waste in Yazd. This descriptive cross-sectional study was conducted on a waste disposal site in Yazd in 2017–2018. sampling of the construction and demolition waste disposal site was performed for 12 months (Jul 2017 to Jul 2018). According to a researcher-made checklist, data on the weight, the density and volume of these wastes were collected. The descriptive statistics tests of data were processed in Excel software. 53,445 t of waste are annually generated in Yazd that the amount of cement and concrete, bricks, tile and ceramic (TC), ferrous metals, non-ferrous metals, glass, plastic, wood, and are approximately 38%, 20%, 14%, 11%, 6%, 5%, 3%, and 3%, respectively. With regards to the high volume of waste generated and a remarkable part of the recyclable waste, urban planners should pay attention to the implementation of waste reduction and recycling programs.

**Specifications table**

**Table:** 

Subject area	Environmental science
More specific subject area	Waste management, Urban management
Type of data	Tables, Figure
Data collection method	Sampling of the construction and demolition waste disposal site was performed one time at week for 12 months from Jul 2017 to Jul 2018. samples were transferred to the School of Public Health and classified into 8 classes base on a researcher-made checklist. then the weight, the density and volume of waste were experimentally calculated. Information about the weight of trucks that entered the disposal site was collected from the municipality.
Data format	Raw/Analyzed
Experimental factors	Composition, weight, density and volume of construction and demolition waste
Experimental features	The waste generated was determined based on the composition.
Data source location	Yazd, Yazd Province, Iran
Data accessibility	The data are available in this article

**Value of the data**

● This study presents a detailed description of the construction and demolition waste generated in a city.
● The data on quantity and quality of construction waste will be very valuable for urban management and development.
● The quantitative and qualitative data will assist policy-makers in managing the reduction, recycling and reuse programs of construction and demolition waste.

## 1 Data

**Fig. 1:**
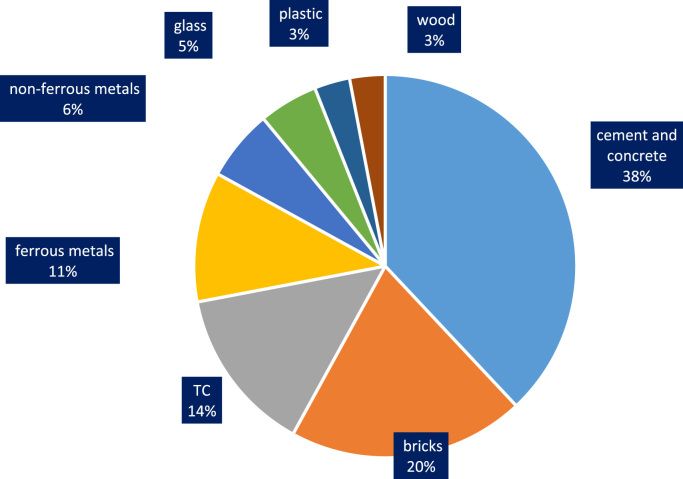
The percentage of cement and concrete, bricks, tile and ceramic (TC), ferrous metals, non-ferrous metals, glass, plastic, wood waste in Yazd.

**Fig. 2:**
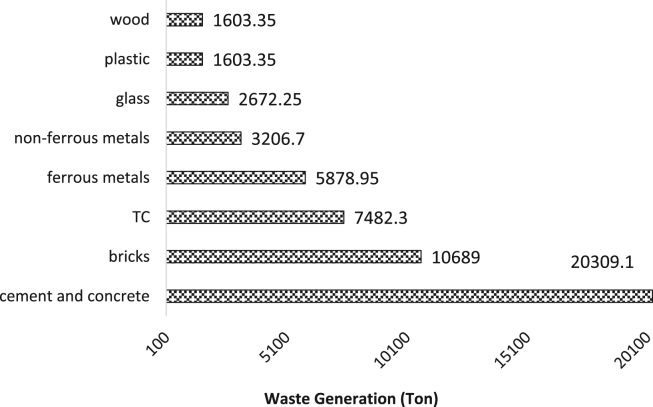
The annual generation of construction and demolition waste in Yazd.

**Fig. 3:**
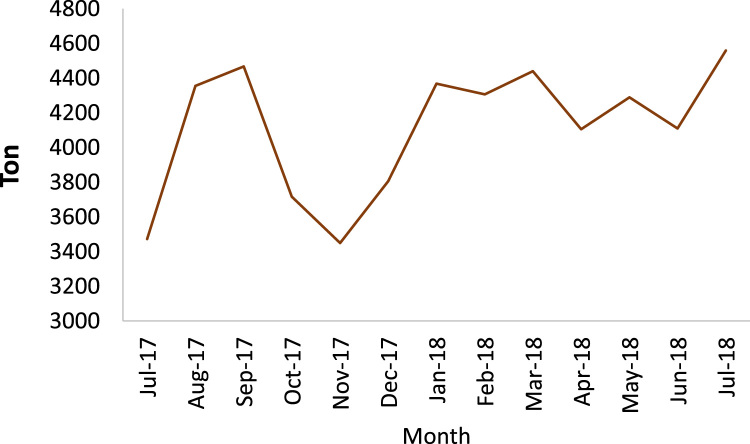
The construction and demolition waste generation change over the study time.

**Fig. 4:**
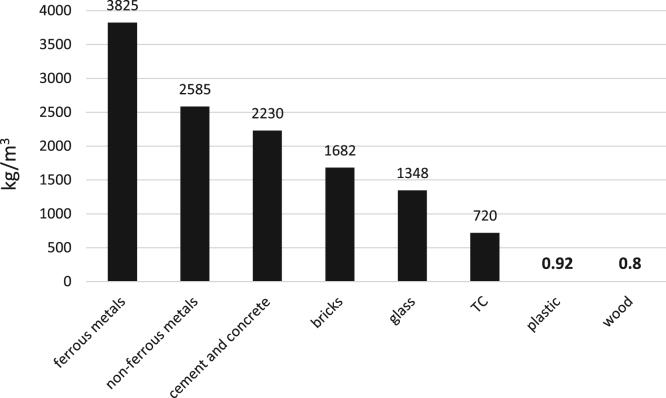
The density of construction and demolition waste in Yazd.

**Fig. 5:**
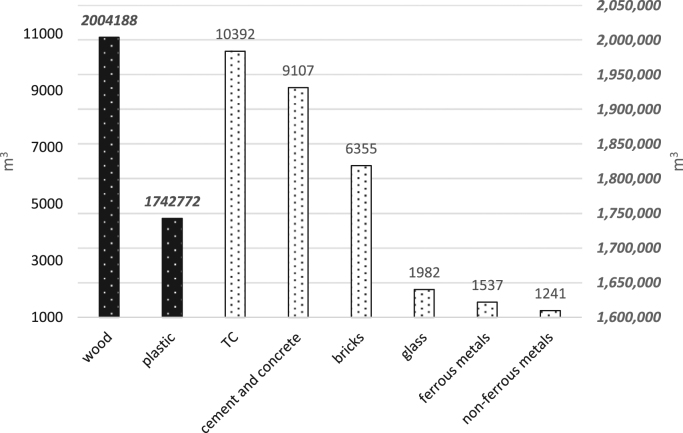
The annual uncompressed volume of construction and demolition waste in Yazd.

Today, with the increasing population of cities and the expansion of construction, demolition and restoration of buildings, the amount of construction and demolition waste is increasing dramatically [1], [2]. The construction and demolition waste is a high volume waste group in municipal solid waste which needs special attention [3]. In terms of quality, construction and demolition waste include materials that are formed during the construction, renovation and demolition of a building [4], [5]. Therefore, it is very important to understand the qualitative and quantitative characteristics of the waste. This paper presents data supporting quantitative and qualitative analysis of construction and demolition waste in Yazd city, Iran. Fig. 1 shows the percentage of construction and demolition waste generated in Yazd. As can be seen from Fig. 1, cement and concrete and wood and plastic are the largest (28%) and smallest (3%) component of construction and demolition waste. According to the results of this study, the total amount of construction and demolition waste generated in Yazd is 53,445 t/y. Fig. 2, Fig. 3, Fig. 4, Fig. 5 show the annual generation, generation change over the study time, density and annual uncompressed volume of the construction and demolition waste in Yazd, respectively.

## 2 Experimental design, materials and methods

### 2.1 Study area description

Yazd city is a desert city in central Iran and the capital of Yazd Province (Fig. 2), which is inscribed on UNESCO׳s World Heritage List as a priceless adobe city and has a total area of approximately 2397 km^2^. The population of Yazd was 656,474 in 2016.

### 2.2 Sample collection and analytical procedures

Yazd has an official site for the disposal of construction and demolition waste. Sampling was done from trucks that entered the disposal site systematically once a week. Samples were transferred to the Laboratory of the School of Public Health and classified into 8 classes based on a researcher-made checklist (cement and concrete, bricks, tile and ceramic (TC), ferrous metals, non-ferrous metals, glass, plastic, wood). Then the weight, density and volume of wastes were experimentally calculated. To calculate the density of each class of the waste, the sample was introduced into a cylindrical container containing water and then, based on the water output from the cylinder (equivalent to the sample volume) and the sample weight, the sample density was calculated. Finally, considering the relative weight of the samples, the overall weight of the trucks and the number of days of the activity landfill site, the annual amount of waste was obtained.
