# Supplementary material for: Quantitative and qualitative analysis of construction and demolition waste in Yazd city, Iran
Source: Data Brief. 2018 Oct 30;21:2622–6. doi: 10.1016/j.dib.2018.10.141 (PMC6290117; doi:10.1016/j.dib.2018.10.141)
Supplement: Supplementary file 1 — Supplementary material. [file mmc1.doc]

Conflict of Interest and Authorship Conformation Form

Please check the following as appropriate:

- All authors have participated in (a) conception and design, or analysis and interpretation of the data; (b) drafting the article or revising it critically for important intellectual content; and (c) approval of the final version.
- This manuscript has not been submitted to, nor is under review at, another journal or other publishing venue.
- The authors have no affiliation with any organization with a direct or indirect financial interest in the subject matter discussed in the manuscript
- The following authors have affiliations with organizations with direct or indirect financial interest in the subject matter discussed in the manuscript:

Author’s name Affiliation

Mohsen Ansari, (1) Student Research Committee, Shahid Sadoughi University of Medical Sciences, Yazd, Iran. (2) Environmental Science and Technology Research Center, Department of Environmental Health Engineering, Shahid Sadoughi University of Medical Sciences, Yazd, Iran.

Mohammad Hassan Ehrampoush, (1) Environmental Science and Technology Research Center, Department of Environmental Health Engineering, Shahid Sadoughi University of Medical Sciences, Yazd, Iran.
